# Supplementary material for: Immune and neural response to acute social stress in adolescent humans and rodents
Source: Transl Psychiatry. 2024 Jul 25;14:306. doi: 10.1038/s41398-024-03008-5 (PMC11272929; doi:10.1038/s41398-024-03008-5)
Supplement: Supplementary file 1 — Supplementary Tables [file 41398_2024_3008_MOESM1_ESM.docx]

| **Table S1: Subject-Level Covariates and Median Fluorescence Index (MFI) Values – Pre-TSST Blood Samples Cultured Without LPS** | | | | | | | | | | | | | | | | | | | | | | | | | | | | | | | | | | | | | | | | | | | | | |
| --- | --- | --- | --- | --- | --- | --- | --- | --- | --- | --- | --- | --- | --- | --- | --- | --- | --- | --- | --- | --- | --- | --- | --- | --- | --- | --- | --- | --- | --- | --- | --- | --- | --- | --- | --- | --- | --- | --- | --- | --- | --- | --- | --- | --- | --- |
| **Sub No.** | **Dx Status** | **Age** | **Sex** | **BMI** | **EGF** | **Eotaxin** | **FGF-2** | **Flt-3L** | **Fractal-kine** | **G-CSF** | **GM-CSF** | **GRO** | **IFNa2** | **IFNg** | **IL-1a** | **IL-1b** | **IL-1RA** | **IL-2** | **IL-3** | **IL-4** | **IL-5** | **IL-6** | **IL-7** | **IL-8** | **IL-9** | **IL-10** | **IL-12P40** | **IL-12P70** | **IL-13** | **IL-15** | **IL-17A** | **IP-10** | **MCP-1** | **MCP-3** | **MDC** | **MIP-1a** | **MIP-1b** | **PDGF-AA** | **PDGF-AB/BB** | **RANTES** | **sCD40L** | **TGF-a** | **TNFa** | **TNFb** | **VEGF** |
| 1 | Psych | 14 | F | 25.2 | 82 | 47 | 17.5 | 31 | 19 | 82.5 | 21 | 1642.2 | 25.5 | 39.5 | 22.5 | 16.5 | 57 | 55.75 | 19.25 | 22 | 30 | 37.5 | 17.5 | 203.5 | 22 | 24.5 | 18 | 15 | 47.5 | 19 | 80.5 | 117.5 | 153.25 | 197 | 622.75 | 25 | 59 | 772.75 | 125.25 | 12000 | 317.5 | 31 | 79.25 | 95 | 21.5 |
| 2 | Psych | 12 | F | 31.9 | 280 | 216.5 | 14.5 | 28 | 19.75 | 20 | 21.75 | 231.5 | 21 | 28 | 31.75 | 63 | 268 | 52.75 | 20 | 19 | 193.5 | 42 | 18.75 | 893 | 27.5 | 24.5 | 22.25 | 27 | 224.5 | 27.5 | 49.5 | 477 | 415 | 363.5 | 101.75 | 116 | 193.25 | 902.5 | 141 | 3922.2 | 84.5 | 61.75 | 150.25 | 217.5 | 28 |
| 3 | Psych | 19 | M | 25.2 | 331 | 304.75 | 40.5 | 34.5 | 40.5 | 77.25 | 59.5 | 277 | 42 | 210.5 | 85.75 | 52 | 293.25 | 100.5 | 34.5 | 40.75 | 176.25 | 79.25 | 42 | 427.25 | 109.75 | 90.5 | 87.25 | 47.25 | 252 | 90.75 | 144 | 2090.2 | 1433.2 | 615.75 | 795 | 262.5 | 123 | 1119.2 | 73.5 | 5610.2 | 110.75 | 129.5 | 115.75 | 321.5 | 52.5 |
| 4 | Psych | 12 | M | 21.9 | 27.5 | 68.5 | 14 | 23.75 | 16.5 | 21.5 | 20 | 153.25 | 19.5 | 20 | 25 | 17.5 | 27.25 | 52 | 15 | 15.5 | 18.75 | 23.25 | 14.75 | 128.25 | 23 | 16.25 | 17.5 | 15 | 22 | 18 | 26.5 | 390.25 | 147.5 | 55.5 | 517.25 | 20 | 27 | 841 | 188 | 2408.2 | 69 | 22.5 | 50.5 | 38 | 21.5 |
| 5 | Psych | 13 | M | 18.4 | 31.5 | 45 | 14.5 | 21.5 | 11.5 | 14 | 17.75 | 48.25 | 15.5 | 18.75 | 19 | 15.75 | 17.5 | 48 | 13.5 | 17.25 | 12.75 | 18 | 10.25 | 34 | 17.5 | 19.25 | 13.5 | 14.25 | 12.25 | 18.25 | 22.75 | 146.5 | 150.25 | 13.5 | 666 | 12 | 23 | 680.25 | 24 | 1518.5 | 33.75 | 20 | 56 | 15.5 | 15 |
| 6 | Psych | 14 | F | 24.5 | 917 | 131.75 | 23 | 37.25 | 28.5 | 35.25 | 31.5 | 7248.2 | 33.5 | 34.5 | 32 | 25 | 86.75 | 56.5 | 22 | 34 | 55.25 | 30 | 30.5 | 124.75 | 31 | 142.25 | 25.5 | 21.75 | 86.5 | 26.5 | 51 | 194.25 | 190 | 182.5 | 292 | 6769 | 61.25 | 8422.2 | 2333.5 | 13905 | 1645.8 | 28 | 54.5 | 87 | 44.25 |
| 7 | Psych | 14 | M | 22.6 | 357 | 79 | 15 | 19 | 18.5 | 59.75 | 17.5 | 10854 | 19.5 | 18.5 | 24.5 | 16 | 17.5 | 48 | 17.5 | 23 | 15 | 21.5 | 23 | 41.25 | 18.5 | 14 | 12.5 | 15 | 26 | 14 | 23.5 | 124 | 264.25 | 21.5 | 318 | 19.25 | 28 | 11826 | 2699 | 15372 | 3333.5 | 12.5 | 48.5 | 21.5 | 23 |
| 8 | Control | 13 | F | 21.7 | 67.25 | 67 | 38 | 42.75 | 20.5 | 33 | 31 | 265.25 | 21 | 113.75 | 35.5 | 205 | 98.25 | 67.5 | 28 | 25 | 29 | 990.5 | 16.5 | 766 | 27 | 39.25 | 27.25 | 35 | 41.5 | 29.75 | 187.5 | 809.5 | 807.5 | 53.5 | 344.25 | 1212.2 | 226.5 | 861 | 240.75 | 2547 | 127.75 | 100.25 | 536.5 | 29 | 56 |
| 9 | Control | 18 | M | 18.2 | 77 | 132.25 | 25.25 | 42 | 14 | 20 | 22 | 63 | 14.25 | 101.5 | 34 | 87.5 | 51 | 63.5 | 34.25 | 18 | 17.25 | 345 | 9.75 | 482 | 22.25 | 26 | 22 | 47.5 | 16 | 20.5 | 152.5 | 902 | 724.25 | 30.5 | 331 | 145 | 463 | 585.5 | 319 | 1172.5 | 55.5 | 44.25 | 280 | 20 | 34.5 |
| 10 | Control | 18 | M | 32.1 | 97.5 | 135.5 | 19.5 | 45 | 19.5 | 20.5 | 19 | 40.5 | 15 | 20.5 | 37.75 | 17 | 24 | 58.75 | 44 | 18 | 14 | 28.5 | 13.25 | 61.25 | 19.75 | 25.5 | 21 | 25.25 | 11.5 | 22 | 39.75 | 745.75 | 2336.8 | 15 | 175.5 | 19 | 39 | 268.25 | 333 | 1005.8 | 44 | 26.5 | 64.5 | 20.5 | 15 |
| 11 | Control | 13 | F | 26.1 | 1145 | 158 | 43.5 | 39.75 | 28.5 | 72 | 39 | 13260 | 34.5 | 67.5 | 39 | 47.5 | 169.5 | 71.5 | 26.5 | 39 | 105.75 | 96.25 | 42.5 | 643.5 | 39 | 28.5 | 45.5 | 38 | 172.75 | 37.5 | 71.25 | 362.75 | 465 | 643.75 | 150.5 | 118 | 183.75 | 14367 | 3520 | 9708 | 4815.5 | 33 | 164.5 | 186 | 63.5 |
| 12 | Control | 18 | F | 24.4 | 1526.5 | 94 | 35 | 27.5 | 237.75 | 57.75 | 34 | 12534 | 54 | 57.5 | 33.5 | 27.5 | 39.75 | 55 | 30 | 33.5 | 23.5 | 55 | 35 | 237.75 | 28 | 21.5 | 21.5 | 29.5 | 31.5 | 21.5 | 70 | 300.25 | 905 | 26.5 | 622.75 | 39.5 | 81 | 12403 | 2698.5 | 12697 | 3396 | 27 | 101.75 | 28 | 43.5 |
| 13 | Psych | 15 | M | 18.1 | 52.25 | 100 | 16.5 | 27 | 32.5 | 17.25 | 18.5 | 50 | 18 | 28.5 | 28 | 14.5 | 22 | 54 | 20 | 15.25 | 12.5 | 25 | 11 | 70.5 | 19.75 | 22.5 | 16.5 | 17 | 13.5 | 18.5 | 47.5 | 422.5 | 496.75 | 16.25 | 547.5 | 21.75 | 37 | 403.75 | 85.5 | 520.25 | 63.25 | 30.5 | 84.25 | 18.5 | 14.5 |
| 14 | Psych | 14 | M | 19.1 | 197 | 87 | 18 | 32.5 | 14.25 | 20.25 | 17 | 28.75 | 20.5 | 57 | 32 | 17.25 | 76.5 | 58 | 20.5 | 17.5 | 29.25 | 22.5 | 11.5 | 133 | 23.5 | 22.75 | 19.25 | 16.5 | 44.25 | 22.5 | 32.5 | 611 | 182.75 | 115 | 439 | 27.5 | 53.75 | 575.5 | 105 | 1197 | 62.5 | 30 | 80.75 | 51.25 | 16 |
| 15 | Psych | 16 | F | 17.4 | 38.75 | 26 | 15 | 22.5 | 16 | 25.25 | 14.5 | 58 | 17 | 25.75 | 24 | 18.5 | 22.5 | 50.5 | 20 | 16.5 | 17 | 21 | 15.5 | 198.75 | 31 | 14 | 15.5 | 15 | 13.25 | 16.5 | 33 | 165.25 | 79.5 | 17 | 471.25 | 19 | 30 | 937.5 | 345 | 4083.5 | 78 | 23 | 46 | 19.5 | 21.5 |
| 16 | Psych | 14 | F | 20.3 | 3445 | 575 | 42.5 | 43 | 42.5 | 77 | 53 | 13849 | 45.5 | 45.5 | 66.5 | 48 | 820.75 | 78.25 | 41 | 47.25 | 590.75 | 53.5 | 52.75 | 893 | 69.5 | 45.25 | 50.5 | 31.5 | 833.25 | 56 | 53 | 314.75 | 739 | 1469 | 859.25 | 349.5 | 346.5 | 17444 | 5194.2 | 13310 | 5802.8 | 35.5 | 96.5 | 877 | 70.25 |
| 17 | Psych | 12 | F | 20.9 | 51 | 32.75 | 15.75 | 25.5 | 22.75 | 19.25 | 24.75 | 25 | 156.5 | 25.75 | 20.5 | 34.75 | 31 | 52.5 | 19 | 20.5 | 15.5 | 65 | 13 | 122.25 | 22.25 | 23.5 | 16 | 17.5 | 16.5 | 17 | 27 | 481 | 284.75 | 28.25 | 966.5 | 74.5 | 129.5 | 495.75 | 24.5 | 1109.8 | 46 | 21.5 | 117.75 | 23.75 | 14 |
| 18 | Psych | 15 | M | 20.6 | 2050 | 848 | 87.25 | 84.25 | 107.5 | 95 | 146 | 5980.5 | 91.5 | 98.5 | 116.25 | 167.5 | 1854.2 | 140.5 | 66.5 | 85.5 | 1162.8 | 1161.8 | 89.5 | 17006 | 162.25 | 105 | 149.5 | 96.25 | 2818.2 | 137.75 | 244 | 512.75 | 10028 | 6597.5 | 436.5 | 769.75 | 679 | 3516.5 | 1375 | 1778.5 | 107.25 | 814.5 | 104.25 | 3451 | 152.75 |
| 19 | Psych | 17 | F | 22.3 | 186 | 40 | 16.5 | 18 | 13.5 | 14.25 | 17.5 | 4788.8 | 19 | 31.25 | 28 | 27.5 | 29.5 | 54 | 17.5 | 22 | 17.75 | 27.5 | 25.5 | 87.5 | 23 | 22 | 25.25 | 12.25 | 29.5 | 27 | 64 | 104.5 | 176.5 | 69 | 120 | 54.5 | 31.75 | 4903 | 2375 | 13844 | 1426.2 | 16 | 25 | 47.5 | 20.5 |
| 20 | Psych | 17 | F | 23.0 | 729.5 | 120.75 | 35.5 | 27.5 | 19.25 | 132 | 36.75 | 6275 | 34 | 40.5 | 31.5 | 22 | 32 | 61 | 28.25 | 27 | 17.75 | 29.5 | 28.75 | 134.5 | 30.5 | 27 | 23 | 26.5 | 24.5 | 25 | 71.5 | 196 | 595.75 | 28.5 | 830 | 37 | 34 | 15780 | 1557.5 | 14178 | 4353.5 | 29.5 | 80 | 31 | 28 |
| 21 | Psych | 15 | M | 37.2 | 835 | 107 | 19 | 30.5 | 17.25 | 35.5 | 19.5 | 7146.5 | 24 | 21 | 34 | 17.5 | 37.5 | 53 | 30.5 | 20 | 21 | 29.5 | 22 | 135 | 20 | 23 | 17 | 18 | 16 | 19.75 | 31.25 | 254.25 | 934.5 | 15.5 | 471 | 23.5 | 68 | 13325 | 6231.5 | 12404 | 5481.8 | 27.5 | 112 | 16.5 | 18.5 |
| 22 | Psych | 12 | F | 38.8 | 353.75 | 53.5 | 18.5 | 19.5 | 18.25 | 53 | 27.5 | 4031.5 | 16.5 | 36.5 | 19.5 | 17.5 | 36.25 | 53.25 | 20.5 | 21.5 | 19 | 22 | 18.5 | 67 | 21 | 19 | 15.5 | 17 | 23.25 | 17 | 42.25 | 109.25 | 233.5 | 41 | 413 | 18.5 | 24 | 7144 | 305 | 14588 | 1954 | 20 | 52.25 | 23.5 | 19 |
| 23 | Psych | 18 | M | 23.5 | 222.5 | 94 | 28 | 29.5 | 22.5 | 41.75 | 28.25 | 6107.8 | 25 | 27 | 31.5 | 23 | 38 | 53 | 22.5 | 28.5 | 27 | 26 | 26 | 65.5 | 25 | 19 | 22.5 | 25 | 31.5 | 22.5 | 43 | 121.75 | 806 | 43 | 331.5 | 27.75 | 39 | 9049 | 1220.8 | 16285 | 968.5 | 21.75 | 50 | 39 | 32.5 |
| 24 | Psych | 13 | F | 18.8 | 175.5 | 60.5 | 44.5 | 27.5 | 24 | 66 | 36.25 | 99 | 20.5 | 190.25 | 29 | 23.5 | 40 | 74.75 | 19.25 | 23.5 | 22.5 | 76 | 33.25 | 254 | 24 | 20.5 | 18.5 | 52.25 | 534 | 18.5 | 207 | 131 | 868.25 | 63.75 | 612.75 | 89 | 83.25 | 1336.5 | 218.75 | 2147.8 | 100 | 53.5 | 61.5 | 21 | 325 |
| 25 | Psych | 13 | F | 28.3 | 2012.8 | 54 | 37.25 | 49.75 | 35 | 91.5 | 39.25 | 9231.2 | 43.5 | 32.5 | 41.75 | 30.5 | 51.75 | 68.5 | 35.5 | 36.25 | 31 | 35.25 | 39 | 76 | 37 | 24.5 | 34.5 | 30.5 | 44.25 | 33.5 | 43.5 | 606.25 | 195 | 83 | 471.5 | 36.5 | 74.5 | 14472 | 5671.2 | 8359.2 | 3260 | 30 | 76.5 | 56.5 | 44.75 |
| 26 | Psych | 16 | M | 24.0 | 939.5 | 222.5 | 24.5 | 28 | 25 | 39 | 27.5 | 4457.2 | 26 | 29 | 53.5 | 47.5 | 229 | 72.25 | 23.5 | 27.25 | 146.25 | 45.5 | 28 | 283 | 58.25 | 50.5 | 48.25 | 22 | 280.75 | 48.75 | 33.25 | 294.75 | 574.5 | 1065.5 | 303.5 | 105 | 112 | 9216 | 2346.5 | 9544 | 2987.5 | 23.5 | 51.5 | 356.25 | 37.5 |
| 27 | Psych | 18 | F | 30.2 | 211 | 32 | 19.5 | 26.75 | 17.25 | 66.25 | 19.5 | 8103.8 | 21.25 | 25.25 | 22.5 | 19.5 | 26 | 47.5 | 19 | 25 | 16 | 35.5 | 22.75 | 221.5 | 17.5 | 14 | 15.5 | 17.5 | 19.5 | 16.75 | 29.5 | 310.75 | 102.5 | 18 | 451.5 | 27 | 52.5 | 7628 | 1332 | 6422.5 | 1075.5 | 20.5 | 92.5 | 23 | 24.25 |
| 28 | Psych | 17 | M | 27.7 | 1599.2 | 58.5 | 21 | 24 | 19.5 | 35 | 21.5 | 6596.5 | 21.5 | 22.5 | 40 | 20.5 | 49.5 | 51.25 | 27 | 25 | 34.5 | 23.5 | 23.5 | 98 | 23.75 | 15.5 | 17 | 19 | 52.25 | 18 | 35.5 | 353.25 | 300 | 81 | 446.75 | 34.25 | 46 | 13001 | 6248.5 | 10412 | 4001.5 | 18 | 65.25 | 70.5 | 30.25 |
| 29 | Psych | 17 | M | 21.8 | 31.5 | 31.25 | 14.25 | 20.5 | 17.25 | 17 | 17.5 | 37.5 | 19 | 24 | 23 | 17.5 | 17.25 | 48.75 | 18.5 | 21 | 16.25 | 19.25 | 15 | 30.5 | 17 | 14.75 | 15.75 | 18.5 | 14.25 | 15.75 | 26.5 | 223 | 49.5 | 15.5 | 213.5 | 17.5 | 27 | 581.75 | 73.25 | 639.5 | 47.75 | 14.5 | 36.5 | 20 | 21.5 |
| 30 | Control | 18 | M | 23.0 | 843.5 | 113 | 21.25 | 22.5 | 24 | 91.5 | 25 | 8562.5 | 26.25 | 20.5 | 47.25 | 45.25 | 44.25 | 69 | 25 | 26.5 | 25 | 56.25 | 28 | 87.5 | 52.75 | 39.75 | 44.75 | 19.25 | 49.25 | 40.25 | 27 | 258.5 | 742.25 | 60.25 | 277.25 | 50 | 38.5 | 9213.2 | 1823.5 | 8053.2 | 2190.8 | 21 | 63.5 | 52.25 | 31.25 |
| 31 | Psych | 14 | F | 24.8 | 32 | 121.75 | 23.5 | 37.5 | 17 | 20.75 | 22.5 | 85 | 25 | 43 | 23.75 | 20.25 | 31.5 | 52 | 17 | 20 | 17.5 | 26.5 | 16.75 | 64.5 | 23.25 | 20.5 | 21.25 | 23.75 | 18.25 | 18.25 | 45 | 351.5 | 397.75 | 21 | 698 | 26 | 37 | 969.25 | 153.5 | 750.5 | 78.25 | 23.5 | 65 | 20.25 | 23.5 |

| **Table S2: Subject-Level Covariates and Median Fluorescence Index (MFI) Values – Pre-TSST Blood Samples Cultured With LPS** | | | | | | | | | | | | | | | | | | | | | | | | | | | | | | | | | | | | | | | | | | | | | |
| --- | --- | --- | --- | --- | --- | --- | --- | --- | --- | --- | --- | --- | --- | --- | --- | --- | --- | --- | --- | --- | --- | --- | --- | --- | --- | --- | --- | --- | --- | --- | --- | --- | --- | --- | --- | --- | --- | --- | --- | --- | --- | --- | --- | --- | --- |
| **Sub No.** | **Dx Status** | **Age** | **Sex** | **BMI** | **EGF** | **Eotaxin** | **FGF-2** | **Flt-3L** | **Fractal-kine** | **G-CSF** | **GM-CSF** | **GRO** | **IFNa2** | **IFNg** | **IL-1a** | **IL-1b** | **IL-1RA** | **IL-2** | **IL-3** | **IL-4** | **IL-5** | **IL-6** | **IL-7** | **IL-8** | **IL-9** | **IL-10** | **IL-12P40** | **IL-12P70** | **IL-13** | **IL-15** | **IL-17A** | **IP-10** | **MCP-1** | **MCP-3** | **MDC** | **MIP-1a** | **MIP-1b** | **PDGF-AA** | **PDGF-AB/BB** | **RANTES** | **sCD40L** | **TGF-a** | **TNFa** | **TNFb** | **VEGF** |
| 1 | Psych | 14 | F | 25.2 | 86.5 | 47 | 17 | 30.5 | 16.5 | 54.5 | 22.5 | 671.5 | 27 | 41.5 | 24.5 | 110.5 | 92.5 | 57 | 21 | 22 | 29.5 | 644.25 | 18.75 | 669.5 | 21.75 | 30.5 | 18 | 16.5 | 46.5 | 20 | 82.5 | 237.5 | 810 | 181 | 632.75 | 504.75 | 1213.2 | 489.75 | 101.5 | 6162 | 154.5 | 35.5 | 481.75 | 89 | 23 |
| 2 | Psych | 12 | F | 31.9 | 306 | 245.75 | 23.5 | 34.75 | 33.5 | 50.5 | 68.5 | 1381 | 35.5 | 37 | 54.5 | 1930.5 | 580.5 | 58.75 | 25 | 33.5 | 202.5 | 2971.2 | 36.5 | 7707.2 | 40 | 64.5 | 33.75 | 39.5 | 250.5 | 34 | 60.5 | 1019.2 | 13846 | 1624.8 | 101 | 8226 | 3278.5 | 1162.5 | 214.5 | 6826 | 131.25 | 91.75 | 2499.5 | 245.5 | 52.75 |
| 3 | Psych | 19 | M | 25.2 | 290 | 245.75 | 39.25 | 32 | 35.75 | 73 | 59.5 | 839.25 | 38.5 | 161.25 | 86.5 | 608.5 | 408 | 97.25 | 30.5 | 39 | 144 | 794.5 | 37.75 | 641.75 | 101 | 87.5 | 85.25 | 44.5 | 215 | 84.5 | 100.5 | 2968.5 | 1782.8 | 523 | 597.5 | 1238.2 | 2211 | 1540.5 | 57.5 | 8503.5 | 144.5 | 124.5 | 1218 | 248.5 | 46 |
| 4 | Psych | 12 | M | 21.9 | 22.5 | 72.5 | 13.5 | 23.5 | 18 | 22 | 22.5 | 289.5 | 20.5 | 21.5 | 25.5 | 233 | 205.5 | 50.5 | 13.5 | 15.75 | 17.5 | 812.5 | 17.25 | 1195.2 | 21.5 | 25.5 | 16.5 | 14.5 | 22 | 19 | 27.5 | 1086.8 | 574.75 | 48 | 520.5 | 423 | 711.25 | 788 | 168.25 | 2135.2 | 54.75 | 42 | 309.5 | 35.75 | 20.75 |
| 5 | Psych | 13 | M | 18.4 | 40 | 41.5 | 17 | 19 | 12.25 | 14 | 18.5 | 83.5 | 15.5 | 19 | 20 | 26.5 | 20.75 | 54 | 14 | 16 | 13 | 75 | 9.5 | 196.5 | 18 | 20 | 14 | 14.5 | 13 | 18.25 | 24.5 | 152.5 | 206 | 14.25 | 731.25 | 40 | 97.5 | 708 | 23 | 1973.2 | 37 | 20.75 | 210.5 | 13.5 | 15.75 |
| 6 | Psych | 14 | F | 24.5 | 204 | 65.5 | 18 | 26.5 | 16 | 218 | 24 | 2875 | 25 | 27 | 32.5 | 24 | 48 | 56.25 | 26.75 | 24 | 27.5 | 82 | 12 | 482.25 | 16 | 116.25 | 19.75 | 18 | 34 | 20.5 | 39 | 125.75 | 201 | 56.25 | 185.75 | 1991 | 107 | 4614.5 | 869.75 | 14013 | 582.25 | 22 | 62 | 38.5 | 25 |
| 7 | Psych | 14 | M | 22.6 | 482 | 114 | 14.5 | 19.5 | 19.5 | 29.5 | 18 | 13092 | 20.5 | 19 | 51.5 | 43 | 22 | 48.5 | 18.25 | 26.5 | 14.5 | 242 | 28 | 385 | 19 | 15.5 | 13.5 | 15 | 30 | 14 | 21.5 | 176 | 349.5 | 21 | 394.75 | 382.25 | 362.25 | 14038 | 4768 | 14952 | 4354 | 12 | 552.5 | 21 | 24 |
| 8 | Control | 13 | F | 21.7 | 79.75 | 70.5 | 39 | 45 | 23.5 | 40 | 31.5 | 393.5 | 23.75 | 111 | 36.5 | 757 | 106.25 | 71 | 29 | 26.75 | 29.25 | 1498.5 | 17 | 1568 | 28.5 | 47.5 | 29 | 36.75 | 42.75 | 32.25 | 171.5 | 709.5 | 1047 | 57 | 338.75 | 1105.2 | 246.25 | 940 | 268.25 | 3147 | 137.25 | 113 | 733.75 | 32 | 56 |
| 9 | Control | 18 | M | 18.2 | 88.5 | 132.25 | 26.5 | 42.75 | 10.25 | 16.25 | 26.5 | 174.25 | 13.25 | 101 | 51 | 914 | 124.75 | 63 | 43 | 20.5 | 17.5 | 3782 | 10 | 1952 | 22.5 | 42 | 28.5 | 46.75 | 18.5 | 21 | 177.25 | 3922.5 | 5091 | 43.25 | 348.25 | 4235.5 | 3242 | 651.75 | 319.75 | 1749 | 62.75 | 55.75 | 1936 | 18.5 | 35 |
| 10 | Control | 18 | M | 32.1 | 71 | 110 | 17.5 | 36.25 | 13.5 | 18.5 | 17.75 | 70 | 18.5 | 21.5 | 46 | 389.5 | 49 | 55.75 | 45.5 | 18.5 | 13 | 797.5 | 17 | 956 | 17.5 | 26.5 | 21 | 20 | 14.5 | 21.25 | 37.5 | 930 | 2071.5 | 14.5 | 149.5 | 1014 | 1470.2 | 165.5 | 63.25 | 813 | 41 | 26.75 | 880.75 | 18 | 15 |
| 11 | Control | 13 | F | 26.1 | 1035.5 | 222.5 | 49 | 48 | 40.25 | 108 | 49.5 | 12965 | 49.25 | 80 | 42.25 | 250.5 | 246.25 | 73 | 36.25 | 46.5 | 114.5 | 931.5 | 54 | 4325.2 | 48.5 | 37.5 | 50.75 | 47 | 194 | 44.5 | 78.25 | 633.25 | 1336.8 | 683.5 | 170.5 | 729.75 | 641 | 13744 | 4193 | 11003 | 4626.5 | 47.5 | 1077.5 | 214.5 | 72.75 |
| 12 | Control | 18 | F | 24.4 | 1488 | 93.5 | 45.75 | 31.5 | 236.75 | 86 | 43.25 | 13779 | 82.75 | 69.5 | 36 | 127.5 | 77 | 56.5 | 37 | 40 | 26.5 | 186 | 39.5 | 665.25 | 34.5 | 26 | 29 | 37 | 29.5 | 26 | 72.5 | 328.25 | 873.25 | 31.25 | 730.5 | 174 | 404.5 | 14208 | 3939.5 | 11888 | 4126.8 | 42 | 385 | 32 | 50.5 |
| 13 | Psych | 15 | M | 18.1 | 50.25 | 94.75 | 20.25 | 30 | 32.5 | 21 | 18.5 | 84.5 | 18.75 | 31 | 33 | 295.5 | 100 | 56 | 22.25 | 18 | 17.5 | 455.5 | 12.5 | 972 | 20.75 | 25.5 | 18.5 | 18.75 | 15.5 | 19 | 47 | 691.25 | 1484.5 | 21 | 586 | 846.75 | 1649 | 464 | 99 | 555.5 | 69.75 | 49.25 | 668.5 | 18.75 | 17 |
| 14 | Psych | 14 | M | 19.1 | 156.75 | 69.75 | 17.5 | 27.5 | 15.5 | 19.5 | 16 | 59.5 | 17.75 | 45.75 | 27.5 | 139 | 95.5 | 53.5 | 20.5 | 17.5 | 23 | 404 | 12.25 | 1084.5 | 20.75 | 23.5 | 16 | 16.5 | 37 | 19.5 | 33 | 741.75 | 1232.2 | 106 | 442.25 | 762 | 897.5 | 505.25 | 79.5 | 1049.5 | 60.25 | 29.5 | 401 | 45.25 | 15.25 |
| 15 | Psych | 16 | F | 17.4 | 30.5 | 29.5 | 15 | 24 | 17.5 | 22.75 | 19.5 | 171.75 | 19.5 | 28.5 | 26 | 47.5 | 36.75 | 53.5 | 18.5 | 19.5 | 17.75 | 177.25 | 20.5 | 5096.5 | 36 | 19 | 16 | 15.5 | 14.5 | 18.5 | 36.75 | 159.5 | 844.5 | 25.5 | 475.75 | 159.5 | 98.25 | 600 | 134.25 | 1101.2 | 35 | 22.5 | 119.25 | 19.75 | 22 |
| 16 | Psych | 14 | F | 20.3 | 3279.2 | 643.5 | 42.25 | 45 | 43.5 | 87 | 47 | 15900 | 42 | 42 | 73.5 | 95.75 | 914 | 74.5 | 45 | 46.75 | 590 | 196.5 | 50 | 1237 | 65.75 | 43.25 | 46.75 | 28 | 805.25 | 53 | 48 | 366.5 | 878.5 | 1500.8 | 919 | 499.75 | 685 | 18318 | 8862 | 13173 | 7021.2 | 35.5 | 460.75 | 985.75 | 69 |
| 17 | Psych | 12 | F | 20.9 | 44.5 | 33.5 | 16 | 31 | 24.75 | 24.25 | 25.5 | 49.25 | 151.25 | 27 | 21 | 65.75 | 30.75 | 49.25 | 19.5 | 22.5 | 18.5 | 97.25 | 16 | 266.25 | 23 | 25 | 19 | 17.5 | 17.5 | 19.5 | 29.5 | 551 | 361.5 | 32 | 1171 | 78 | 108 | 668.25 | 29 | 2690.2 | 60.5 | 22 | 146.5 | 23.25 | 16.25 |
| 18 | Psych | 15 | M | 20.6 | 2141 | 892.5 | 94.5 | 80.25 | 108.25 | 172.25 | 253 | 17075 | 105.5 | 114.75 | 136.5 | 341 | 2184.5 | 138.25 | 63 | 97 | 1206.2 | 13188 | 99.5 | 24189 | 174 | 119 | 147.5 | 106.5 | 2993.5 | 141.5 | 277 | 737.5 | 23380 | 7183 | 2394 | 871.75 | 759 | 3702.2 | 1382 | 395.5 | 101.75 | 863.75 | 480 | 3449.8 | 176 |
| 19 | Psych | 17 | F | 22.3 | 159.5 | 40 | 17 | 18.5 | 16.25 | 32.25 | 19 | 5473.8 | 18.25 | 33.5 | 41 | 29.75 | 29.5 | 55.5 | 16.5 | 23 | 18.75 | 30.5 | 23 | 160.25 | 26 | 22.5 | 26 | 15.5 | 30 | 26 | 63 | 100.5 | 243.5 | 67 | 129.5 | 56.5 | 42 | 6772.8 | 2139.5 | 12187 | 1291.5 | 16.75 | 84 | 45 | 22 |
| 20 | Psych | 17 | F | 23.0 | 1457 | 208 | 36 | 30.25 | 18 | 89 | 42.5 | 6531.2 | 34.75 | 51.5 | 34.5 | 163.75 | 45 | 58.25 | 27 | 24.75 | 18 | 377.25 | 27.5 | 915.5 | 36 | 33.5 | 24.5 | 23.5 | 25.25 | 31.5 | 91.25 | 306.25 | 901.5 | 35 | 1085.5 | 1160.5 | 232.25 | 18845 | 2809.5 | 11719 | 6508.5 | 37 | 669.5 | 34 | 37 |
| 21 | Psych | 15 | M | 37.2 | 1202.5 | 115.5 | 20 | 27.5 | 18.5 | 30.5 | 18.5 | 8418.5 | 26.5 | 19.75 | 43 | 53.5 | 65 | 55.5 | 44 | 22 | 20 | 417.5 | 23.5 | 1334.5 | 21.5 | 25 | 16 | 16 | 19 | 19.5 | 31 | 316.25 | 1610.5 | 14.5 | 483.75 | 757.25 | 934.25 | 14107 | 6763.2 | 14208 | 6705.2 | 34.5 | 950.25 | 16.5 | 19 |
| 22 | Psych | 12 | F | 38.8 | 548 | 56.5 | 21.25 | 22 | 19.5 | 43.5 | 30.5 | 5980.2 | 20 | 38 | 25 | 20 | 40.25 | 57.5 | 28 | 25 | 20 | 27.5 | 20.5 | 163.75 | 24.75 | 22 | 16.5 | 18.5 | 23.5 | 17.5 | 50.5 | 138.5 | 271.5 | 49 | 426 | 25.75 | 39 | 8654.5 | 630.5 | 17346 | 2894.8 | 21.5 | 115.5 | 25 | 21.25 |
| 23 | Psych | 18 | M | 23.5 | 374.75 | 147.25 | 36 | 34 | 27 | 83.25 | 42.5 | 10371 | 31 | 32 | 43 | 168 | 64 | 54.5 | 22.75 | 36 | 30 | 1249.5 | 38.5 | 2250.2 | 28.5 | 23.5 | 27 | 28.5 | 38.5 | 25.5 | 50 | 316 | 1925 | 59 | 460 | 2874.2 | 1417.8 | 13536 | 3784.2 | 17548 | 1712 | 23.5 | 1607.2 | 45 | 40.75 |
| 24 | Psych | 13 | F | 18.8 | 152.75 | 61 | 49.25 | 24.5 | 26 | 141 | 39.75 | 591 | 25.75 | 177 | 35.5 | 641.25 | 112.75 | 72.5 | 19.5 | 25 | 23.5 | 949.25 | 36.5 | 1974.8 | 26 | 39 | 20.5 | 56.75 | 368.5 | 20.75 | 156.5 | 193.5 | 1226.8 | 52.75 | 638.5 | 592.5 | 1899.5 | 2363 | 240.75 | 3369.2 | 178.5 | 62.5 | 362.5 | 24 | 230.75 |
| 25 | Psych | 13 | F | 28.3 | 1906.5 | 51.75 | 37.25 | 48.5 | 37 | 77 | 39 | 9592 | 42.5 | 31.5 | 41 | 33 | 58 | 65.75 | 33.5 | 35.5 | 29 | 96.5 | 41.75 | 376 | 37 | 26.5 | 35.25 | 30 | 43 | 35.5 | 42 | 621.5 | 214.5 | 87 | 488.75 | 110 | 227 | 14581 | 5416 | 8852.5 | 3096.2 | 30 | 343 | 55.5 | 44 |
| 26 | Psych | 16 | M | 24.0 | 1304.5 | 235.25 | 23.75 | 29.25 | 25 | 44.25 | 30.75 | 4251.5 | 27 | 28.75 | 51 | 57.5 | 247.75 | 74.5 | 24 | 28.75 | 154.5 | 98.5 | 27 | 359.75 | 62.5 | 54 | 50 | 21 | 287.5 | 48.5 | 34 | 391 | 673.5 | 1128 | 339.25 | 139 | 168.5 | 9323 | 4697.8 | 9548.2 | 3040.5 | 24 | 146 | 370 | 40 |
| 27 | Psych | 18 | F | 30.2 | 223.75 | 33.5 | 18.5 | 27.5 | 19.5 | 64.5 | 23 | 8509.5 | 23 | 24 | 23 | 33 | 32 | 48 | 19.5 | 26.5 | 17 | 245 | 26.5 | 1651.5 | 18 | 19 | 17 | 17.5 | 19 | 17 | 30.75 | 337.5 | 160.75 | 19.5 | 451.25 | 358 | 316 | 7842 | 1345.2 | 6507.5 | 1120.8 | 23.5 | 297.25 | 23.25 | 27 |
| 28 | Psych | 17 | M | 27.7 | 1742.5 | 59.75 | 20.5 | 26 | 20.5 | 36.5 | 24.5 | 6173.5 | 25 | 22.75 | 42 | 40 | 68.25 | 54.5 | 29.25 | 23 | 35 | 251.25 | 28 | 750 | 24 | 19 | 18 | 19.5 | 50 | 21.5 | 35.25 | 663.5 | 1226 | 84 | 384.75 | 288.5 | 663.5 | 13040 | 7224.5 | 9846.8 | 4006 | 22.5 | 358.75 | 67.75 | 29 |
| 29 | Psych | 17 | M | 21.8 | 34.75 | 32 | 13 | 20.75 | 17.25 | 17 | 17.5 | 58.5 | 18.5 | 23 | 21 | 86.25 | 32 | 47.5 | 18.25 | 21.5 | 15 | 232.75 | 15.25 | 961.5 | 17 | 16.25 | 15.5 | 19 | 12 | 15.5 | 27 | 316 | 379.5 | 16 | 224.25 | 428.25 | 796 | 559.25 | 70.25 | 656 | 44.5 | 16.25 | 364.25 | 20 | 20 |
| 30 | Control | 18 | M | 23.0 | 1281.8 | 150.75 | 28 | 28.5 | 29 | 52 | 44 | 11775 | 37.5 | 27.5 | 69.5 | 217.75 | 92.5 | 76.5 | 26.5 | 34.5 | 29 | 1176 | 34.5 | 1221.8 | 68.5 | 52 | 50 | 25 | 63 | 52.75 | 34.5 | 1999.2 | 4944 | 92.5 | 301 | 2180.5 | 1102 | 11822 | 3024 | 7293.2 | 3301 | 26.25 | 997.5 | 63 | 42.75 |
| 31 | Psych | 14 | F | 24.8 | 37.5 | 139.25 | 22 | 40.25 | 24 | 24 | 25.5 | 257 | 26.5 | 43 | 28 | 240.75 | 149 | 54 | 20.25 | 24 | 17.5 | 446.75 | 20.5 | 805.5 | 22.75 | 28.25 | 25.5 | 26 | 19.25 | 20 | 42.25 | 549.5 | 455.5 | 22 | 777.5 | 279 | 781.25 | 1169.5 | 190.25 | 1461 | 103.75 | 32.5 | 316.5 | 21.5 | 28 |

| **Table S3: Subject-Level Covariates and Median Fluorescence Index (MFI) Values – Post-TSST Blood Samples Cultured Without LPS** | | | | | | | | | | | | | | | | | | | | | | | | | | | | | | | | | | | | | | | | | | | | | |
| --- | --- | --- | --- | --- | --- | --- | --- | --- | --- | --- | --- | --- | --- | --- | --- | --- | --- | --- | --- | --- | --- | --- | --- | --- | --- | --- | --- | --- | --- | --- | --- | --- | --- | --- | --- | --- | --- | --- | --- | --- | --- | --- | --- | --- | --- |
| **Sub No.** | **Dx Status** | **Age** | **Sex** | **BMI** | **EGF** | **Eotaxin** | **FGF-2** | **Flt-3L** | **Fractal-kine** | **G-CSF** | **GM-CSF** | **GRO** | **IFNa2** | **IFNg** | **IL-1a** | **IL-1b** | **IL-1RA** | **IL-2** | **IL-3** | **IL-4** | **IL-5** | **IL-6** | **IL-7** | **IL-8** | **IL-9** | **IL-10** | **IL-12P40** | **IL-12P70** | **IL-13** | **IL-15** | **IL-17A** | **IP-10** | **MCP-1** | **MCP-3** | **MDC** | **MIP-1a** | **MIP-1b** | **PDGF-AA** | **PDGF-AB/BB** | **RANTES** | **sCD40L** | **TGF-a** | **TNFa** | **TNFb** | **VEGF** |
| 1 | Psych | 14 | F | 25.2 | 81 | 42.5 | 15.5 | 28 | 15 | 48.25 | 19 | 550.5 | 21 | 36.25 | 20.5 | 18.5 | 54.5 | 55 | 18.5 | 18.75 | 30 | 60 | 15.25 | 250 | 19.5 | 24 | 16.5 | 14 | 42.5 | 17.5 | 75.25 | 111.25 | 144.25 | 180.25 | 553.75 | 33 | 112.5 | 447.5 | 79.75 | 5194 | 136 | 26.5 | 95 | 89.5 | 18 |
| 2 | Psych | 12 | F | 31.9 | 277.75 | 224 | 15.75 | 26.75 | 20.25 | 20 | 21.5 | 189.75 | 19 | 26.75 | 31.5 | 52.5 | 261.25 | 53.5 | 16.5 | 19.25 | 185 | 38.25 | 19 | 841.5 | 27 | 23 | 22 | 29.75 | 227 | 27.25 | 49.25 | 403.25 | 429.5 | 381.5 | 91.5 | 114.25 | 166 | 740.25 | 119 | 3119.5 | 74 | 57.5 | 132.75 | 222 | 28 |
| 3 | Psych | 19 | M | 25.2 | 274.5 | 228 | 35.5 | 31.75 | 33 | 60 | 54 | 63.5 | 36.5 | 152.5 | 77.75 | 47.5 | 217.25 | 95.75 | 31 | 38.5 | 141 | 66 | 36 | 323 | 95.5 | 77 | 78.5 | 44 | 201 | 72.75 | 101.5 | 1089 | 1185.5 | 478.75 | 511.5 | 193.5 | 94.75 | 658 | 49.5 | 1591 | 62 | 105.5 | 95.5 | 225.5 | 44 |
| 4 | Psych | 12 | M | 21.9 | 32 | 63 | 13 | 22 | 6.5 | 23.25 | 18.75 | 127.75 | 24 | 19.5 | 26 | 19 | 27.5 | 52 | 16 | 16.5 | 18 | 22.25 | 18 | 54 | 15.75 | 15.5 | 18 | 15 | 21.5 | 18.5 | 27.25 | 407.75 | 66.5 | 54 | 486.5 | 17.75 | 26.25 | 1014.5 | 62.5 | 2715.2 | 51.5 | 21.5 | 32 | 38.5 | 19.25 |
| 5 | Psych | 13 | M | 18.4 | 32.5 | 39.5 | 14.5 | 18 | 12 | 14.5 | 18 | 288 | 13.5 | 18.5 | 19 | 14.75 | 17.5 | 48 | 15 | 15 | 12.75 | 17.75 | 10.5 | 33.5 | 17.75 | 19 | 13 | 14.5 | 13 | 17 | 22.5 | 154 | 117.5 | 13.5 | 652.75 | 12 | 21 | 1001 | 29 | 3011 | 45.75 | 18 | 51 | 14.25 | 14.5 |
| 6 | Psych | 14 | F | 24.5 | 1041.5 | 170.5 | 22.5 | 39 | 29 | 20.5 | 32 | 11623 | 33 | 40 | 30.75 | 23.5 | 97.25 | 58.5 | 18.5 | 31 | 58.5 | 29 | 17.5 | 142.75 | 28.25 | 154.75 | 24.5 | 20.5 | 93.5 | 25 | 65.5 | 217.5 | 156.5 | 223 | 385.25 | 10946 | 69.5 | 11319 | 5526.8 | 16588 | 2735.2 | 26.75 | 56.5 | 97.5 | 44.5 |
| 7 | Psych | 14 | M | 22.6 | 309.25 | 57.5 | 15.5 | 18 | 18.75 | 43.25 | 17 | 9641 | 19.5 | 21 | 23 | 15.75 | 19 | 46.5 | 19.5 | 23 | 15.5 | 21 | 24.25 | 38 | 16.5 | 14.5 | 13 | 15 | 26 | 15 | 24.25 | 93.25 | 198 | 15.5 | 288.75 | 19 | 26.5 | 11151 | 1796.5 | 13440 | 2511.5 | 15 | 45.75 | 20 | 23 |
| 8 | Control | 13 | F | 21.7 | 79 | 71 | 44.5 | 46.5 | 24 | 36.75 | 30 | 129.25 | 25.5 | 120.5 | 39 | 311.75 | 107.5 | 70.25 | 29.5 | 26.75 | 33.5 | 1435.5 | 17 | 1129.2 | 33 | 44 | 31.75 | 41 | 47 | 31.25 | 198.5 | 890.25 | 1268 | 61.5 | 326 | 2383 | 369.5 | 567.75 | 190 | 1256 | 90.5 | 111.25 | 782 | 30.5 | 66 |
| 9 | Control | 18 | M | 18.2 | 64.75 | 132 | 21.25 | 32.5 | 12.5 | 18 | 18.5 | 39.25 | 13.5 | 78.75 | 27.75 | 16.5 | 27 | 58 | 29.5 | 16.5 | 15.5 | 50 | 10 | 110.75 | 19 | 22 | 18 | 28.5 | 15 | 21.5 | 102.5 | 688.5 | 419.5 | 24.75 | 330.25 | 29.25 | 52.5 | 475.5 | 251.5 | 654.5 | 47.75 | 35 | 116.5 | 18 | 24 |
| 10 | Control | 18 | M | 32.1 | 78 | 141 | 18.25 | 39.25 | 17.5 | 16.75 | 18 | 42.75 | 12.25 | 22 | 37 | 15.5 | 21 | 58.5 | 47.75 | 19 | 14.25 | 27 | 13.5 | 56 | 16.5 | 25 | 22.25 | 21 | 13 | 21.5 | 40 | 613 | 1720.8 | 15.5 | 189.5 | 17.5 | 40.5 | 176.5 | 142.5 | 908 | 39.25 | 23 | 48.5 | 19 | 12 |
| 11 | Control | 13 | F | 26.1 | 1268.2 | 175.75 | 42 | 40 | 30.25 | 58.75 | 41.25 | 12208 | 38.5 | 65.25 | 38.5 | 39 | 170 | 70.5 | 33.25 | 39 | 106.5 | 47.5 | 43 | 623.5 | 43 | 31.75 | 48 | 35.75 | 169.5 | 37.75 | 73.75 | 310.5 | 723 | 645 | 154.25 | 107 | 114.25 | 14239 | 4285 | 10774 | 4745.5 | 36 | 114 | 199 | 63 |
| 12 | Control | 18 | F | 24.4 | 1541.8 | 112 | 41.5 | 27 | 226.5 | 52.75 | 35.25 | 13716 | 96 | 60.5 | 32 | 69.5 | 52 | 55 | 31 | 36.25 | 25.25 | 115 | 35.5 | 483.5 | 31 | 22.5 | 24 | 31.75 | 27.5 | 23.5 | 64.5 | 317.25 | 960 | 29 | 766.5 | 88.5 | 238 | 14065 | 3104.2 | 11260 | 3606 | 28.25 | 180.5 | 29 | 46 |
| 13 | Psych | 15 | M | 18.1 | 48 | 87.75 | 18 | 23.25 | 33 | 15.75 | 17.5 | 48.75 | 14.5 | 27.75 | 26.5 | 23 | 25.5 | 53.5 | 19.5 | 15.5 | 15.5 | 41.5 | 9.75 | 169 | 17.5 | 21.5 | 14.25 | 18.5 | 11.5 | 17 | 47.5 | 338 | 488.25 | 16 | 570.5 | 36 | 106.75 | 364.5 | 122.25 | 456 | 55 | 30.75 | 133.5 | 16 | 14.5 |
| 14 | Psych | 14 | M | 19.1 | 201.5 | 84.5 | 20.75 | 30.5 | 15.5 | 22.5 | 16 | 47.75 | 22.5 | 57 | 29.25 | 23.75 | 78.5 | 56.75 | 22.25 | 18.5 | 31.75 | 45.5 | 14 | 192.5 | 22.5 | 24 | 18 | 17 | 46.5 | 21 | 34.5 | 626 | 275 | 119 | 503.5 | 34 | 101.5 | 721.75 | 131.5 | 1800.8 | 76.75 | 28.5 | 98 | 56.25 | 17.5 |
| 15 | Psych | 16 | F | 17.4 | 26.5 | 24.75 | 14 | 22 | 15 | 21.5 | 16.5 | 66 | 17.25 | 27 | 22.5 | 18.75 | 20 | 53 | 18 | 16 | 18 | 19.25 | 15 | 112.5 | 35.25 | 12 | 14.5 | 15 | 13.75 | 15.5 | 34.5 | 165 | 142.25 | 17 | 491 | 18.5 | 31.75 | 477.25 | 114.5 | 2040 | 25 | 16 | 48 | 17.5 | 20 |
| 16 | Psych | 14 | F | 20.3 | 3740 | 613.5 | 42 | 41 | 42.5 | 77 | 46 | 16045 | 40 | 41 | 63 | 41.25 | 816.25 | 75.25 | 47 | 48.75 | 576.25 | 46.5 | 51.5 | 826.25 | 66.25 | 39.5 | 46.5 | 33 | 814 | 52 | 50 | 275 | 804.75 | 1414.5 | 871.25 | 333.5 | 356 | 18502 | 7274.8 | 13106 | 6894.5 | 31 | 98.5 | 923.5 | 67.5 |
| 17 | Psych | 12 | F | 20.9 | 44 | 33.5 | 15.25 | 26 | 25.5 | 25 | 22 | 47.5 | 156.5 | 25 | 21 | 39.5 | 29.5 | 52.5 | 21 | 23.5 | 16.75 | 67 | 15.5 | 149.75 | 23 | 24.25 | 16.75 | 17.75 | 17.5 | 19.5 | 28.25 | 501.5 | 323.5 | 29.75 | 879 | 102.5 | 139.75 | 578.5 | 25 | 1992 | 60.5 | 20.5 | 145.75 | 23.5 | 16 |
| 18 | Psych | 15 | M | 20.6 | 2079.8 | 826 | 87 | 78 | 95.5 | 73.25 | 117.5 | 480.5 | 79.25 | 99.75 | 110 | 107.5 | 1851.8 | 137.5 | 64 | 73.5 | 1209 | 100.75 | 65.75 | 4170 | 152.75 | 80.25 | 138.5 | 86 | 2866.2 | 131 | 250 | 390 | 1009.8 | 6344 | 413.75 | 777.25 | 656.75 | 3751 | 1286 | 1982.8 | 100.25 | 844.5 | 106 | 3287.5 | 141 |
| 19 | Psych | 17 | F | 22.3 | 365.5 | 48 | 21.75 | 20 | 20.25 | 29 | 21.5 | 8397 | 20 | 32.75 | 36 | 40 | 34.5 | 59.5 | 18.5 | 26 | 21 | 34 | 23.5 | 108.5 | 41.5 | 30 | 32.5 | 18.75 | 42.5 | 37.5 | 62 | 112.75 | 296.75 | 83.5 | 157 | 78.75 | 37 | 10189 | 2781.5 | 10630 | 2878.5 | 19.5 | 34 | 55.5 | 27 |
| 20 | Psych | 17 | F | 23.0 | 857 | 110.25 | 33.75 | 26.5 | 19 | 92.5 | 35 | 5475.2 | 29 | 38.75 | 30.5 | 20 | 32 | 57.25 | 25.5 | 25 | 16 | 27 | 22 | 118.5 | 29 | 26 | 23 | 22 | 24 | 24 | 65.75 | 165 | 583 | 27 | 813.25 | 32 | 29 | 14412 | 1134 | 13294 | 4275 | 28 | 69.25 | 27 | 30 |
| 21 | Psych | 15 | M | 37.2 | 546 | 66 | 22.5 | 24.5 | 20 | 31.25 | 19 | 4669.2 | 25 | 22 | 33.5 | 19 | 27.5 | 53.5 | 33 | 19 | 18.5 | 23 | 19.5 | 56.5 | 22 | 23 | 17.5 | 18.5 | 19.5 | 19 | 34 | 207 | 635.5 | 14 | 350.5 | 18.5 | 36 | 9112.5 | 3121.5 | 11759 | 3229.8 | 26.75 | 74.25 | 15 | 18.5 |
| 22 | Psych | 12 | F | 38.8 | 343 | 54.5 | 22 | 22 | 19.5 | 50.5 | 32.5 | 3488 | 23.75 | 38.5 | 24 | 19.5 | 44.5 | 55.75 | 24.25 | 25 | 19.5 | 24 | 21 | 78 | 24.5 | 21.5 | 17.25 | 18.75 | 24 | 18.5 | 50 | 173.25 | 331.25 | 49 | 515.25 | 20.75 | 26.5 | 6108.8 | 309 | 17226 | 1848 | 23 | 56.25 | 26.5 | 21 |
| 23 | Psych | 18 | M | 23.5 | 308.75 | 144.5 | 31 | 27 | 22 | 47 | 29.5 | 10146 | 22 | 26.5 | 32 | 24 | 41.5 | 53 | 20.5 | 28.5 | 29 | 24 | 27.5 | 86.25 | 23.5 | 17.5 | 21.5 | 22.5 | 36.5 | 21.5 | 45 | 138 | 876.75 | 56 | 481.75 | 30.5 | 42 | 14022 | 3299.2 | 16624 | 1580.8 | 19 | 51.5 | 42.5 | 30.5 |
| 24 | Psych | 13 | F | 18.8 | 179.25 | 61 | 37.75 | 23 | 27.5 | 31.5 | 35.25 | 51 | 17.5 | 201 | 27.5 | 22.75 | 40.5 | 66.25 | 19.5 | 20.5 | 21.5 | 70.5 | 41.25 | 284 | 22 | 18.75 | 17.5 | 43.5 | 505.75 | 17 | 210 | 161.5 | 876 | 49.25 | 671.25 | 88.5 | 69.5 | 760.5 | 195.5 | 1340 | 59.25 | 54.5 | 58 | 19 | 308.5 |
| 25 | Psych | 13 | F | 28.3 | 2161.5 | 50.5 | 37.5 | 41.5 | 38.5 | 68.75 | 41 | 9523.5 | 42 | 30.5 | 39.75 | 27 | 49.5 | 67 | 39.5 | 36.5 | 30.5 | 37 | 39 | 84.5 | 35.5 | 24 | 33 | 29.5 | 41.5 | 32 | 39 | 518.5 | 192.5 | 74.75 | 567.25 | 36.5 | 73.75 | 15033 | 6125.2 | 9680.5 | 3454.2 | 28.5 | 71 | 55.75 | 45.5 |
| 26 | Psych | 16 | M | 24.0 | 948.5 | 199 | 24 | 27.5 | 23.5 | 40.25 | 28 | 4267.2 | 26.5 | 30 | 51.5 | 47.5 | 210 | 74.5 | 24.25 | 27.5 | 131 | 43.25 | 24.25 | 257 | 59 | 50.5 | 45.5 | 20.5 | 250.5 | 46.75 | 32.5 | 270.25 | 593.5 | 988.5 | 284.5 | 97.25 | 104 | 8506 | 2440 | 9801 | 2935.2 | 21.5 | 48.75 | 301.25 | 37 |
| 27 | Psych | 18 | F | 30.2 | 269.25 | 32 | 18.5 | 25.5 | 18 | 41 | 20 | 8372.8 | 21.5 | 25.5 | 23.5 | 24.5 | 26.5 | 48 | 18 | 26.25 | 16 | 83.25 | 24 | 677.25 | 18 | 15 | 16.5 | 18.5 | 17.5 | 17.5 | 29.5 | 387.5 | 130 | 20 | 439.75 | 61.5 | 102.25 | 8444.5 | 1357 | 5512 | 1155.5 | 18.75 | 127 | 22.5 | 26.5 |
| 28 | Psych | 17 | M | 27.7 | 996.5 | 46.5 | 19.5 | 21 | 16.25 | 33 | 18 | 6099 | 19.5 | 21.5 | 35.5 | 17.5 | 38 | 50.5 | 30 | 23 | 25.5 | 20.5 | 25.5 | 72 | 19.75 | 14.25 | 14 | 17 | 36.5 | 16.5 | 34.5 | 268.5 | 244 | 57.25 | 334 | 26.25 | 40.5 | 11256 | 4894 | 10128 | 2782 | 16 | 51.75 | 52.25 | 24.5 |
| 29 | Psych | 17 | M | 21.8 | 29 | 30.5 | 13 | 19 | 15.5 | 16.25 | 15 | 55.25 | 16.5 | 22 | 19 | 16.5 | 14.75 | 46.5 | 16 | 17.75 | 13.5 | 18 | 13 | 29.5 | 14.75 | 11.5 | 13.5 | 16.25 | 12.5 | 14 | 26.5 | 222.75 | 56.25 | 14.5 | 223.75 | 15.5 | 26 | 616.25 | 78.75 | 820.5 | 52.5 | 12 | 37 | 19 | 18.5 |
| 30 | Control | 18 | M | 23.0 | 1083.5 | 123 | 23.5 | 23 | 22.5 | 37.25 | 25 | 10423 | 26 | 22 | 51.5 | 47.75 | 48.5 | 70 | 24.75 | 28 | 26 | 40.75 | 27 | 82.5 | 56 | 42.25 | 41.5 | 21 | 58.5 | 44.25 | 27 | 217.5 | 709.75 | 67 | 257 | 57 | 36 | 10656 | 2301 | 6522.8 | 2881.8 | 21 | 52 | 55.75 | 32.5 |
| 31 | Psych | 14 | F | 24.8 | 40.75 | 146.5 | 20.75 | 35 | 19.75 | 21.5 | 22.5 | 146.5 | 23 | 42 | 27 | 20 | 28 | 51.25 | 19 | 21.5 | 17.5 | 27.75 | 17.75 | 74.5 | 22 | 20.5 | 20.5 | 26 | 18 | 19.5 | 41.75 | 478 | 455.75 | 22 | 772.5 | 27.5 | 42 | 2084.5 | 310.5 | 1469.8 | 121 | 22.75 | 67.5 | 18.75 | 25.25 |

| **Table S4: Subject-Level Covariates and Median Fluorescence Index (MFI) Values – Post-TSST Blood Samples Cultured With LPS** | | | | | | | | | | | | | | | | | | | | | | | | | | | | | | | | | | | | | | | | | | | | | |
| --- | --- | --- | --- | --- | --- | --- | --- | --- | --- | --- | --- | --- | --- | --- | --- | --- | --- | --- | --- | --- | --- | --- | --- | --- | --- | --- | --- | --- | --- | --- | --- | --- | --- | --- | --- | --- | --- | --- | --- | --- | --- | --- | --- | --- | --- |
| **Sub No.** | **Dx Status** | **Age** | **Sex** | **BMI** | **EGF** | **Eotaxin** | **FGF-2** | **Flt-3L** | **Fractal-kine** | **G-CSF** | **GM-CSF** | **GRO** | **IFNa2** | **IFNg** | **IL-1a** | **IL-1b** | **IL-1RA** | **IL-2** | **IL-3** | **IL-4** | **IL-5** | **IL-6** | **IL-7** | **IL-8** | **IL-9** | **IL-10** | **IL-12P40** | **IL-12P70** | **IL-13** | **IL-15** | **IL-17A** | **IP-10** | **MCP-1** | **MCP-3** | **MDC** | **MIP-1a** | **MIP-1b** | **PDGF-AA** | **PDGF-AB/BB** | **RANTES** | **sCD40L** | **TGF-a** | **TNFa** | **TNFb** | **VEGF** |
| 1 | Psych | 14 | F | 25.2 | 78.5 | 44.75 | 17.5 | 28 | 16.5 | 39 | 22.5 | 947 | 24 | 39 | 24.5 | 188.5 | 110 | 55 | 19 | 21 | 29 | 996.25 | 18 | 897.75 | 22 | 33 | 16.5 | 16.5 | 44.5 | 19 | 80 | 373.25 | 1916.5 | 179.5 | 622.5 | 1184.5 | 2106.8 | 533.25 | 90.25 | 7546.8 | 145.5 | 38.5 | 683 | 87 | 21 |
| 2 | Psych | 12 | F | 31.9 | 309 | 244.25 | 21 | 34.5 | 30.75 | 35.5 | 66.5 | 1181.5 | 34.25 | 37 | 48.5 | 1508.2 | 505.25 | 59.5 | 23.5 | 28.5 | 213 | 2495.8 | 35.5 | 7179.8 | 34.5 | 55.5 | 32.75 | 37.25 | 251.5 | 32.5 | 59.75 | 856.25 | 12435 | 1313.5 | 99.5 | 7293 | 2850.5 | 932.5 | 179 | 5220.5 | 113 | 88.5 | 2329 | 240.5 | 53.5 |
| 3 | Psych | 19 | M | 25.2 | 236 | 185.5 | 34.5 | 30.25 | 28.75 | 58.5 | 47.75 | 335.75 | 27.5 | 136 | 70.5 | 504 | 317 | 89.5 | 28.5 | 38.25 | 123.25 | 813.75 | 12.25 | 560.25 | 82.5 | 78.5 | 73 | 40.5 | 169 | 74.75 | 86 | 2531.5 | 1701.8 | 400.25 | 497 | 1458.5 | 2075.8 | 967.5 | 52.5 | 5101.5 | 83.25 | 105.5 | 1234 | 196.25 | 42 |
| 4 | Psych | 12 | M | 21.9 | 28 | 80.5 | 16.5 | 26.5 | 19.75 | 28.5 | 31 | 337.25 | 23 | 29 | 36.5 | 263.25 | 271.25 | 53.75 | 16 | 20 | 20.5 | 1649 | 18.5 | 1252.8 | 28.5 | 24.5 | 20 | 18 | 24 | 21.5 | 34.5 | 7144 | 2051.2 | 64 | 562.5 | 1887.8 | 1827 | 723 | 175 | 2170 | 60 | 58 | 699.75 | 42 | 27 |
| 5 | Psych | 13 | M | 18.4 | 29.5 | 38.25 | 16 | 20 | 11 | 14.5 | 17 | 52.5 | 17 | 18 | 19.5 | 50.75 | 28.75 | 49.5 | 14.5 | 18 | 13.25 | 127.5 | 11.5 | 316.75 | 19 | 21 | 14.5 | 16.25 | 13 | 17 | 23.5 | 172.75 | 144 | 14 | 783 | 58.5 | 237.25 | 603.5 | 24.25 | 1119 | 42 | 20.75 | 235 | 16 | 14 |
| 6 | Psych | 14 | F | 24.5 | 489.75 | 109.75 | 23 | 32.75 | 28 | 39.5 | 33 | 7477.8 | 22.25 | 36 | 34 | 73.5 | 96.5 | 59 | 23.5 | 33 | 43.5 | 593.5 | 29.5 | 943.5 | 27.5 | 139.75 | 24.5 | 22 | 62.5 | 26 | 48 | 517.5 | 558.25 | 126.5 | 280.25 | 7521 | 593 | 8252.8 | 1987.5 | 14100 | 1744 | 31.5 | 897 | 62.5 | 41 |
| 7 | Psych | 14 | M | 22.6 | 428.25 | 86.5 | 15 | 19 | 19.5 | 62 | 18 | 12970 | 20 | 19.5 | 25.5 | 35.5 | 20.25 | 49 | 18 | 25 | 15.5 | 132.25 | 28 | 198.25 | 20.5 | 16.5 | 12.5 | 16 | 31.25 | 16 | 23.5 | 143.25 | 278.5 | 15 | 412.5 | 191.75 | 189 | 14648 | 3504.5 | 15681 | 4211 | 12.5 | 417.5 | 18.5 | 24.5 |
| 8 | Control | 13 | F | 21.7 | 69.5 | 68.25 | 35 | 38 | 22.75 | 34.5 | 28.75 | 245.5 | 23 | 106.75 | 35.25 | 1240.5 | 97 | 66.5 | 27 | 27 | 28 | 2580.2 | 14.75 | 2428 | 27.5 | 50 | 29 | 37 | 44 | 26.5 | 189 | 683 | 2053 | 55.25 | 340.25 | 2161.5 | 361 | 613 | 199 | 1479.2 | 91.5 | 113.75 | 1099.5 | 28 | 56 |
| 9 | Control | 18 | M | 18.2 | 73.25 | 109.75 | 23.5 | 36.25 | 14.75 | 24 | 20.25 | 208 | 17.5 | 84.5 | 50 | 1564.5 | 170.75 | 62 | 31.5 | 19.5 | 17.25 | 4467 | 13.25 | 2333.5 | 21.5 | 39 | 27.5 | 37.5 | 18.5 | 22 | 120.25 | 6197.2 | 6898.5 | 58.5 | 278 | 5855.5 | 3883 | 533.25 | 247.5 | 1199 | 59.25 | 51.5 | 2494.8 | 18 | 32 |
| 10 | Control | 18 | M | 32.1 | 69 | 143 | 17.5 | 38 | 14.5 | 21 | 19 | 78 | 16.75 | 21.5 | 43 | 488.25 | 62 | 54.25 | 30.5 | 17.5 | 13 | 1166.5 | 13 | 1297.8 | 17.5 | 32 | 21.75 | 20 | 14.75 | 19.5 | 36.75 | 1459 | 3318.2 | 17.5 | 171 | 1890.5 | 2110.2 | 269.25 | 112.75 | 650 | 58.5 | 26.25 | 1151 | 19.5 | 15.5 |
| 11 | Control | 13 | F | 26.1 | 696.25 | 127.5 | 29 | 29.75 | 21.25 | 53.75 | 37.75 | 9314.8 | 26.75 | 43.25 | 49.5 | 517 | 126.25 | 63.5 | 27 | 32 | 40 | 2802.2 | 20 | 6966.5 | 39 | 43.25 | 35 | 21 | 61 | 26.75 | 59 | 514.75 | 484 | 232.5 | 152.5 | 1919.8 | 932.5 | 9519.2 | 4379.5 | 18616 | 2591.5 | 33.5 | 184.75 | 83.25 | 31 |
| 12 | Control | 18 | F | 24.4 | 1852.5 | 96.75 | 45.75 | 30.25 | 244.5 | 43.25 | 39.5 | 14184 | 75.25 | 62.25 | 34.5 | 184 | 78.5 | 56.5 | 31.5 | 38.5 | 26 | 277.25 | 39 | 785 | 34.5 | 26 | 25 | 36 | 29 | 23.5 | 69.25 | 369.25 | 1004 | 28 | 767.75 | 376.75 | 669.75 | 14710 | 3841 | 12354 | 4229.5 | 42 | 546.75 | 30.25 | 47.5 |
| 13 | Psych | 15 | M | 18.1 | 43 | 79.75 | 18 | 25.5 | 28.5 | 17.75 | 18 | 88.75 | 16.5 | 26 | 29.5 | 286.75 | 127.5 | 55 | 18.5 | 17.5 | 15 | 387.25 | 12.5 | 987.25 | 19.5 | 26 | 18.5 | 16.25 | 14 | 19 | 46 | 460 | 1280 | 18.5 | 567.25 | 1188.2 | 2024 | 366 | 114 | 482.5 | 57.5 | 49.75 | 738.75 | 17.75 | 16.25 |
| 14 | Psych | 14 | M | 19.1 | 206.75 | 86.25 | 19.5 | 30.25 | 17 | 23 | 18.5 | 88 | 21 | 51.75 | 33 | 222.25 | 140.5 | 59 | 23.5 | 19 | 26 | 708 | 15.5 | 1635.2 | 23.25 | 29.5 | 19 | 19.5 | 39.75 | 20 | 37 | 713 | 2701.5 | 144.5 | 379.25 | 1398.8 | 1926 | 607.25 | 122 | 1609.8 | 69.75 | 34.25 | 656 | 45.5 | 16 |
| 15 | Psych | 16 | F | 17.4 | 24.5 | 27.5 | 18 | 22.25 | 17.75 | 20.5 | 16.75 | 156 | 16 | 33 | 23.25 | 23 | 27 | 50.5 | 16.5 | 17.5 | 21.75 | 72 | 17 | 2033 | 48.25 | 13 | 16.25 | 17 | 14 | 16.25 | 43 | 212.5 | 608.5 | 21 | 517 | 96.75 | 65.5 | 479.75 | 118.75 | 2265.2 | 25.5 | 16.25 | 92 | 19 | 22 |
| 16 | Psych | 14 | F | 20.3 | 4146.5 | 685.5 | 47 | 46.5 | 47 | 90.25 | 52.75 | 16742 | 49.75 | 48.75 | 70.75 | 89.5 | 927.25 | 81 | 49.5 | 50.5 | 633 | 283.5 | 59.75 | 1341 | 73 | 50 | 51 | 32 | 864.5 | 59.75 | 57.25 | 397.5 | 1065.8 | 1590 | 1040.8 | 748.75 | 899 | 19396 | 7948.8 | 14473 | 7791.5 | 40.5 | 688.25 | 1003.5 | 75 |
| 17 | Psych | 12 | F | 20.9 | 44.75 | 30.5 | 16 | 28 | 24 | 22.75 | 23.5 | 50.5 | 143.25 | 27 | 19.5 | 37.25 | 27 | 52.5 | 18 | 22.75 | 15 | 62.5 | 13.75 | 159.25 | 23 | 22.5 | 16.5 | 17 | 16.5 | 18 | 25 | 566.5 | 314 | 28.75 | 983.5 | 46 | 72.5 | 621.5 | 26 | 2494 | 56 | 21.5 | 122.75 | 25.25 | 15 |
| 18 | Psych | 15 | M | 20.6 | 2172.5 | 907.25 | 96.75 | 93 | 125.25 | 279.25 | 328 | 19286 | 129 | 132.5 | 168 | 562 | 2195.8 | 160.25 | 66.75 | 120.25 | 1212 | 17428 | 145.75 | 24178 | 184.25 | 174 | 167.5 | 122.5 | 2946.5 | 151 | 272 | 751.75 | 23428 | 8044.5 | 2570.5 | 1289.2 | 889.5 | 3850.5 | 1427.5 | 351.75 | 119.25 | 915.5 | 1126.8 | 3535 | 194.5 |
| 19 | Psych | 17 | F | 22.3 | 370.25 | 51.5 | 25.5 | 21 | 22 | 62.25 | 22 | 8864 | 22 | 37 | 37.5 | 49.5 | 40 | 62.5 | 20.75 | 27.5 | 21 | 57.5 | 26.5 | 235.5 | 44 | 32.75 | 35 | 20 | 43.5 | 38 | 74.5 | 139.5 | 329 | 85.25 | 152 | 104.5 | 69 | 11047 | 3190 | 10200 | 3197.5 | 21.75 | 163.25 | 57 | 29 |
| 20 | Psych | 17 | F | 23.0 | 610.75 | 99.75 | 30.75 | 25.25 | 20.25 | 317.25 | 38 | 6078.5 | 34 | 38.25 | 32.5 | 89.25 | 33.5 | 57 | 26 | 26.5 | 17 | 146.25 | 26 | 428 | 30.75 | 26.5 | 23 | 23.5 | 24.5 | 24.5 | 71.75 | 164.75 | 577 | 24 | 676.25 | 238.5 | 83.5 | 13849 | 1200.2 | 15314 | 4014.5 | 29 | 305 | 27.5 | 28 |
| 21 | Psych | 15 | M | 37.2 | 957.75 | 73.5 | 25 | 28 | 21.5 | 40.5 | 20.5 | 6543 | 27 | 23.75 | 39.5 | 32 | 37 | 55.5 | 35.75 | 23 | 18.5 | 219.5 | 23 | 591.75 | 22.5 | 25 | 19 | 19 | 27.5 | 22 | 37 | 220.75 | 824.75 | 16.5 | 419 | 488 | 447.25 | 12015 | 4077.8 | 12476 | 4672.2 | 31 | 732.25 | 17.25 | 21.25 |
| 22 | Psych | 12 | F | 38.8 | 381 | 48 | 20.75 | 19.5 | 18.5 | 76.5 | 28.5 | 4049.5 | 19.25 | 37 | 19.5 | 20.5 | 36.5 | 52 | 22.75 | 23.5 | 19.5 | 29.75 | 18 | 274 | 23.25 | 19.75 | 16 | 17 | 22.5 | 17.25 | 42 | 144.75 | 321.25 | 39 | 363.5 | 34.5 | 39.5 | 6747.5 | 389.75 | 16948 | 1907.5 | 20 | 111.75 | 23 | 19 |
| 23 | Psych | 18 | M | 23.5 | 147 | 86 | 28 | 29 | 25.5 | 50.75 | 35 | 5795 | 28 | 29 | 34 | 136.25 | 47 | 55 | 26 | 30 | 25.5 | 883 | 30.5 | 1131.5 | 26 | 22 | 24.5 | 24 | 31 | 24 | 43.75 | 181.75 | 923.25 | 41.75 | 280.75 | 2352.5 | 885.25 | 8458.2 | 1147.5 | 16686 | 765.5 | 23 | 1586 | 38.5 | 37.5 |
| 24 | Psych | 13 | F | 18.8 | 166.25 | 69.5 | 43.75 | 25.75 | 23.5 | 106 | 40.25 | 644.5 | 24.5 | 196 | 41.75 | 926 | 150.75 | 82.75 | 22.5 | 28.5 | 25 | 1371.2 | 35.25 | 3465.2 | 26.5 | 54.5 | 22 | 51 | 389.75 | 20.5 | 160.5 | 351.75 | 1852.2 | 54.75 | 794 | 1088.5 | 2447 | 2294.5 | 263.5 | 2938.5 | 177.75 | 67 | 454.75 | 24.75 | 235 |
| 25 | Psych | 13 | F | 28.3 | 2150 | 49.5 | 36 | 38.5 | 33.5 | 57.25 | 37.5 | 10350 | 42 | 28.5 | 38 | 32.25 | 59 | 67.5 | 33 | 36.5 | 30.25 | 137.5 | 39 | 513.5 | 35.25 | 24.5 | 30.75 | 30.25 | 38.5 | 31.25 | 39 | 524.75 | 172 | 70 | 473 | 92 | 250 | 14460 | 5515 | 9579.8 | 3380.5 | 29 | 281.25 | 50.25 | 43.5 |
| 26 | Psych | 16 | M | 24.0 | 1249 | 173.5 | 21.5 | 26 | 22 | 45.25 | 25 | 4156.5 | 25 | 27.5 | 48.5 | 54 | 188.5 | 70.5 | 24 | 27 | 115 | 78 | 23 | 283.5 | 54.5 | 45.5 | 40 | 20 | 231.75 | 43.5 | 30 | 242 | 560.5 | 893.5 | 262 | 101 | 121.5 | 8351.8 | 4333.8 | 10090 | 2912.5 | 20 | 97 | 280.75 | 35 |
| 27 | Psych | 18 | F | 30.2 | 302.5 | 40 | 21 | 29.75 | 25 | 286.5 | 36 | 9771.5 | 26.5 | 29.5 | 26 | 135 | 59 | 50.5 | 22.5 | 32.25 | 17 | 1098.5 | 34.75 | 5406 | 22.5 | 27 | 20.5 | 20.5 | 20.25 | 19.5 | 33.5 | 546.75 | 1787.2 | 70 | 428 | 3588 | 1378.5 | 8659.2 | 1817.5 | 6789.8 | 1300.5 | 30.25 | 734 | 24.25 | 36 |
| 28 | Psych | 17 | M | 27.7 | 1038 | 47.5 | 20 | 24 | 20 | 33 | 20.5 | 5349.2 | 25.25 | 22.5 | 37 | 37 | 51 | 53 | 26.25 | 24 | 29 | 274.75 | 24 | 653.5 | 22.5 | 17.5 | 16.75 | 19 | 40.5 | 19 | 34 | 464.25 | 749.5 | 63 | 338.5 | 276.25 | 552.25 | 10672 | 3939.5 | 9449.2 | 2672.8 | 19 | 357.5 | 54.25 | 29 |
| 29 | Psych | 17 | M | 21.8 | 33.25 | 33.75 | 15 | 20 | 17 | 22.5 | 18 | 157.5 | 18.75 | 25.25 | 22 | 103 | 34 | 49.5 | 19 | 20 | 14.5 | 230.5 | 17 | 936.75 | 17.75 | 16.5 | 15 | 20 | 14.5 | 16 | 28.5 | 311 | 435.25 | 16.5 | 234.25 | 637.5 | 990 | 831.25 | 112 | 1454 | 65 | 17 | 365.25 | 20.25 | 22.5 |
| 30 | Control | 18 | M | 23.0 | 1134.8 | 137.5 | 28.5 | 26.5 | 27.5 | 48 | 44 | 11199 | 35.5 | 27 | 67 | 163.75 | 88.5 | 76 | 27 | 35 | 31.5 | 1053.8 | 36 | 774.5 | 66.5 | 54.5 | 52.75 | 26.75 | 65.75 | 52.5 | 32.5 | 2088.5 | 4389.5 | 85.5 | 289 | 1911.2 | 946 | 11003 | 3046.8 | 6744 | 3114.2 | 26 | 963.5 | 62 | 40.5 |
| 31 | Psych | 14 | F | 24.8 | 54.5 | 144.5 | 24 | 37.75 | 26 | 30.75 | 36 | 406.5 | 33 | 46 | 30 | 427 | 251.5 | 56.5 | 26 | 32.5 | 19 | 1021.2 | 25.5 | 2207.2 | 27 | 41 | 25.5 | 30 | 21.75 | 21.75 | 43.5 | 877.75 | 1254.2 | 25.5 | 731.25 | 1646.8 | 1985.8 | 2363.8 | 420 | 2059 | 159.5 | 47 | 590 | 24.25 | 32 |
